# Elevated intracranial pressure after head trauma can be suppressed by antisecretory factor—a pilot study

**DOI:** 10.1007/s00701-020-04407-5

**Published:** 2020-05-22

**Authors:** Kliment Gatzinsky, Ewa Johansson, Eva Jennische, Merna Oshalim, Stefan Lange

**Affiliations:** 1 Department of Neurosurgery, Sahlgrenska University Hospital, SE-413 45 Gothenburg, Sweden; 2 Department of Infectious Diseases, Institute of Biomedicine, University of Gothenburg, Gothenburg, Sweden; 3 Region Västra Götaland, Department of Clinical Microbiology, Sahlgrenska University Hospital, Gothenburg, Sweden

**Keywords:** Traumatic brain injury, Brain edema, Intracranial hypertension, Antisecretory factor, Salovum®, AF-16 ELISA

## Abstract

**Background:**

Control of intracranial pressure (ICP) is a key element in neurointensive care for directing treatment decisions in patients with severe traumatic brain injury (TBI). The anti-inflammatory protein antisecretory factor (AF) has been demonstrated to reduce experimentally induced high ICP in animal models. This report describes the first steps to investigate the uptake, safety, and influence of AF for reduction of elevated ICP in patients with TBI in a clinical setting.

**Method:**

Four patients with severe TBI (Glasgow Coma Scale < 9) that required neurointensive care with ICP monitoring due to signs of refractory intracranial hypertension were investigated. One hundred milliliters of Salovum®, a commercially available egg yolk powder with high contents of AF peptides, was administrated either via nasogastric (patients 1 and 2) or rectal tube (patients 2, 3, and 4) every 8 h for 2 to 3 days as a supplement to the conventional neurointensive care. ICP was registered continuously. Plasma levels of AF were measured by enzyme-linked immunosorbent assay (ELISA) to confirm that Salovum® was absorbed appropriately into the bloodstream.

**Results:**

In the first two patients, we observed that when delivered by the nasogastric route, there was an accumulation of the Salovum® solution in the stomach with difficulties to control ICP due to impaired gastric emptying. Therefore, we tested to administer Salovum® rectally. In the third and fourth patients, who both showed radiological signs of extensive brain edema, ICP could be controlled during the course of rectal administration of Salovum®. The ICP reduction was statistically significant and was accompanied by an increase in blood levels of AF. No adverse events that could be attributed to AF treatment or the rectal approach for Salovum® administration were observed.

**Conclusions:**

The outcomes suggest that AF can act as a suppressor of high ICP induced by traumatic brain edema. Use of AF may offer a new therapeutic option for targeting cerebral edema in clinical practice.

## Introduction

One of the common causes of the bad outcome in patients exposed to severe closed head trauma is the development of cerebral edema with brain swelling and intracranial hypertension which can cause herniation, finally leading to irreversible brain injury and death [15, 26]. Reducing intracranial pressure (ICP) in a timely manner is a critical step in neurointensive care and forms the premise for appropriate secondary damage control and successful clinical outcome and rehabilitation of these patients.

Neuroinflammation plays an important role following traumatic brain injury (TBI) [5, 7, 12, 23]. The mechanisms that drive TBI lesion development and secondary injury due to neuroinflammation, as well as those that promote repair, are complex and multifaceted [7]. Understanding the inflammatory mechanisms that underlie the pathologic outcomes in TBI has attracted increasing interest due to the need of the development of adequate therapeutics to reduce posttraumatic inflammation and brain edema formation [12, 14]. Anti-inflammatory agents have, however, failed to improve TBI outcomes in clinical trials [23]. This report describes the potential role of the endogenous antisecretory and anti-inflammatory protein antisecretory factor (AF) to suppress an elevated ICP in patients suffering from severe TBI with cerebral edema. AF is part of the innate immune system and has been shown to normalize the water and ion transport across the cell membrane [19], to reduce high tissue pressure in tumors [2], and to lower pathologically elevated ICP after focal brain injury in experimental animal models [1, 6, 11]. Four patients are presented within the context of a pilot study representing a first attempt to investigate the uptake, safety, and effect of AF on elevated ICP after severe TBI in a clinical setting.

## Methods

### Patients

Adult patients (18–65 years of age) with severe TBI (Glasgow Coma Scale < 9) admitted to the neurointensive care unit (NICU) at Sahlgrenska University Hospital with signs of intracranial hypertension were included. The patients needed mechanical ventilation and ICP monitoring. Surgical evacuation of intracranial hematoma did not preclude study inclusion; however, patients that had undergone a decompressive craniectomy were excluded. Other exclusion criteria were egg allergy; bilaterally fixed, dilated pupils; brain stem injury; penetrating head injury; and previously known intracranial disease such as tumor, vascular malformation, and hydrocephalus.

ICP was continuously monitored using an intraparenchymal microtransducer (Raumedic Neurovent-P or Spiegelberg™), or an external ventricular drain (EVD) inserted intracranially through a burr hole ventral to the coronal suture over the left or right frontal lobe. Treatment in the NICU setting followed recommended standard protocols for management of severe TBI [3]. The goal was to maintain ICP at levels < 20 mm Hg, with a cerebral perfusion pressure > 60 mm Hg. The study protocol was approved by the local ethics committee in compliance with the Declaration of Helsinki and national law (Dnr. 597-15). Relatives of the patients gave written informed consent for study inclusion.

### AF administration

The spray dried egg yolk powder Salovum®, which has been classified as food for specific medical purposes by the European Union, is the only commercially available form of AF for clinical use. Salovum® has only been utilized for peroral administration in awake patients mainly suffering from various forms of intestinal, inflammatory disease [10, 27, 28]. In the present study, Salovum® administration was initiated if ICP was elevated above levels of 20 mm Hg for more than 1 h. The treatment was supplied for a minimum of 2 days as an add-on therapy to the conventional NICU management with optimized ventilation, sedation, analgesia, and maintenance of fluid balance. If indicated, seizure and infection prophylaxis, and thiopental infusion with continuous EEG monitoring to verify that the treatment was provided at the desired intensity level, was initiated.

Four sachets (16 g) of Salovum® were dissolved in 100 ml of room temperature water. The solution was delivered via the indwelling nasogastric tube or a rectal tube which was inserted 10 cm into the rectum. The rectal tube that was used (ConvaTec Flexi-Seal®, Reading, UK) has a balloon that was lightly inflated to avoid external leakage. Directly after instillation of the full volume of Salovum® into the rectum, the sond was clamped at the entrance to the anus for a minimum of 4 h to avoid backflow. In this way, the conditions for effective absorption were optimized. Salovum® was administered every 8 h during two (patients 1, 2, and 3) to three (patient 4) days. The dosage of Salovum® and the interval for administration were determined based on experiences from previous human trials for oral administration of Salovum® [10, 27].

### Plasma AF analyses

To confirm that Salovum® was absorbed appropriately, blood samples were taken immediately before (control) and 30 min after the start of Salovum® administration in order to measure AF concentration in plasma. Two methods were used:

1. AF-16 was detected by the use of a commercial AF-16 enzyme-linked immunosorbent assay (ELISA) kit (BMA Biomedical, Angst, Switzerland).

2. AF is a constituent of the proteasome. Active AF forms a complex, called compleasome, with complement factor C3c [21]. The level of compleasome was analyzed with a sandwich ELISA as previously described [21]. A monoclonal antibody against proteasome 20Sα6 was used as catching antibody (Enzo Life Sciences, Farmingdale, NY, USA) and a polyclonal antibody against C3c was used as detecting antibody (Agilent Dako, item A0062, Santa Clara, CA, USA). The secondary antibody, alkaline phosphatase–conjugated goat anti-rabbit IgG, was obtained from Jackson ImmunoResearch Europe, Ely, UK.

### Statistics

Student’s *t* test was used for analysis of difference between ICP values before and after Salovum® administration, and paired (samples) *t* test was used for analysis of the ELISA absorbance values. For both analyses, a *p* value < 0.05 was considered significant. AF-16 ELISA is performed as an inhibition method; therefore, the values shown are presented as inverted numbers.

## Results

The patients are presented in chronological order from the first inclusion in May 2016 (patient 1) to the last follow-up in April 2019 (patient 4).

### Patient no. 1 (Fig. 1a)

A 25-year-old man was involved in a skateboard accident without wearing a helmet—GCS 8 prior to intubation. The initial computed tomography (CT) scan showed a thin acute subdural hematoma over the right cerebral hemisphere and bi-frontal contusions which were most prominent on the right side. The patient received a Raumedic Neurovent-P intraparenchymal microtransducer displaying ICP values <20 mm Hg.

**Fig. 1:**
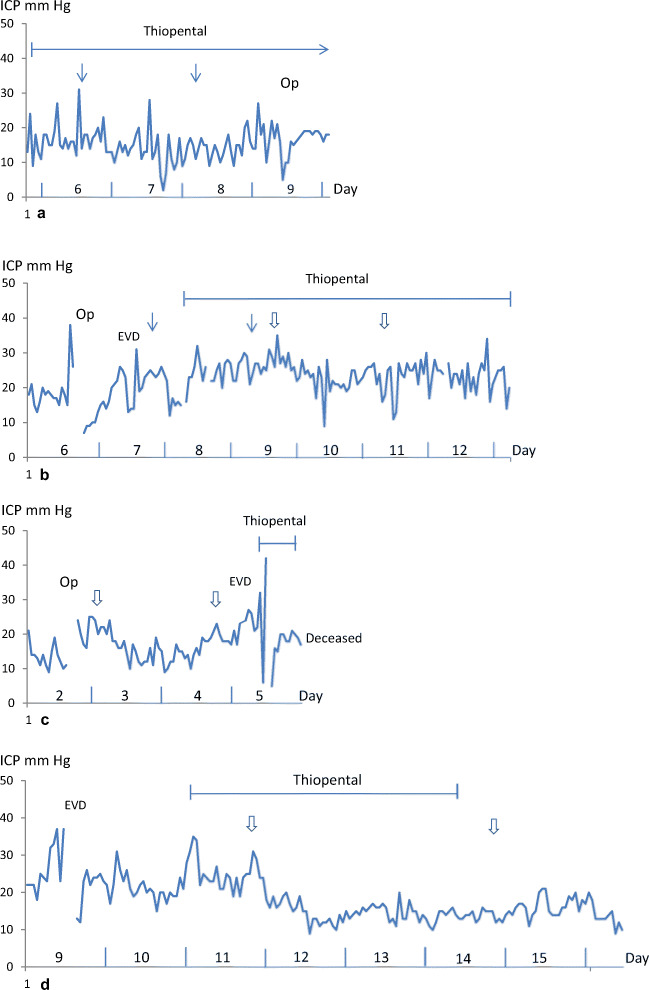
Intracranial pressure (ICP) in the course of nasogastric and rectal Salovum® administration in the four presented patients (**a**–**d**). Thin arrows in **a** and **b** (patients 1 and 2) denote the first and last nasogastric Salovum® delivery. Thick arrows in **b**, **c**, and **d** (patients 2, 3, and 4) denote the first and last rectal delivery. EVD (external ventricular drain) designates insertion of an intraventricular catheter. Op designates operation (type of operation is specified in “Results”)

On day 4 after the trauma, ICP started to rise to levels ≥ 20 mm Hg for extended periods of time. CT showed a slight progress of the contusions with surrounding edema but without any need for surgical intervention. Treatment with thiopental was initiated with ICP still peaking at levels ≥ 20 mm Hg from time to time. Therefore, administration of Salovum® via the nasogastric tube was started on day 6 after the trauma. The patient received a total of six doses in 48 h with ICP being kept at levels < 20 mm Hg for most of the time of Salovum® administration (Fig. 1a). However, due to gastroparesis, there was a gradual increase in gastric fluid volume after administration of Salovum® which was documented by aspiration via the nasogastric tube. The gastric retention was stressful to the patient and caused temporary ICP elevation when Salovum® was given. The nasogastric AF treatment did not result in a statistically significant reduction in ICP (Table 1).

**Table 1:** ICP response to administration of Salovum® based on hourly registrations from the four included patients

**Nasogastric administration**				
**Patient**	**Time period**	**Hours**	**ICP (mean ± SEM)**	***p*value after vs*.*before (*t*test)**
Patient 1	Before Salovum	20–0	17.0 ± 1.0	-
	After Salovum	0–20	15.0 ± 0.7	0.36
	After Salovum	20–40	15.7 ± 2.7	0.57
	After Salovum	40–60	14.2 ± 0.7	0.21
Patient 2	Before Salovum	20–0	19.7 ± 1.2	-
	After Salovum	0–20	21.8 ± 1.1	0.15
	After Salovum	20–40	22.8 ± 0.6	0.0002
**Rectal administration**				
**Patient**	**Time period**	**Hours**	**ICP (mean ± SEM)**	***p*value after vs. before (*t*test)**
Patient 2	Before Salovum	20–0	25.9 ± 0.7	-
	After Salovum	0–20	24.9 ± 1.2	0.45
	After Salovum	20–40	22.8 ± 0.6	0.02
	After Salovum	40–60	22.9 ± 1.2	0.03
	After Salovum	60–80	23.3 ± 0.9	0.06
Patient 3*	Before Salovum	10–0	22.7 ± 1.5	-
	After Salovum	0–10	16.6 ± 1.1	< 0.0002
	After Salovum	10–20	13.3 ± 1.0	< 0.0001
	After Salovum	20–30	13.2 ± 0.7	< 0.0001
	After Salovum	30–40	18.6 ± 0.8	0.0088
Patient 4	Before Salovum	20–0	25.0 ± 1.0	-
	After Salovum	0–20	17.8 ± 1.0	< 0.0001
	After Salovum	20–40	13.7 ± 0.5	< 0.0001
	After Salovum	40–60	13.8 ± 0.5	< 0.0001
	After Salovum	60–80	14.1 ± 0.3	< 0.0001

*In patient 3, the time interval for ICP registration after the external fixation of the tibial fracture until the start of rectal Salovum® administration was 10 h (cf. Fig. 1c). Accordingly, in this patient, the subsequent time intervals for comparison of mean ICP values over time were adapted to the length of this initial period of time.

After Salovum® administration had been terminated, ICP started to rise gradually and became difficult to control on day 9 (Fig 1a). CT showed progression of the right frontal contusion causing a mass effect. The contusion was evacuated with a subsequent reduction of ICP to levels < 20 mm Hg which were sustained. The patient was discharged from the NICU 18 days after the trauma and went to rehabilitation. At 1-year control, he was living on his own, showing no physical sequelae, and planned to start studying at the university.

#### Lessons learned

Administration of Salovum® via a nasogastric tube may not be an optimal means for AF delivery in mechanically ventilated, critically ill patients due to gastroparesis with retention of the solution in the stomach. The retention was stressful to the patient and most likely impeded the uptake of AF. The outcome implicated that blood levels of AF need to be measured to verify an appropriate gastrointestinal absorption of Salovum®.

### Patient no. 2 (Fig. 1b)

A 49-year-old man who was under alcohol intoxication had fallen at home, hitting the back of his head—GCS 7 prior to intubation. The initial CT scan showed bilateral frontal and temporal contusions without exerting a mass effect, and widespread traumatic subarachnoid hemorrhage. A thrombocytopenia was detected and treated instantly. ICP monitoring via a Raumedic Neurovent-P microtransducer showed initial ICP values < 20 mm Hg.

On day 6, ICP peaked at levels above 35 mm Hg (Fig 1b). CT demonstrated progression of the right-sided temporal and frontal contusions which were evacuated with a transient ICP reduction. Intermittent CSF drainage via an EVD could not keep ICP at levels below 20 mm Hg. Administration of Salovum® via the indwelling nasogastric tube was therefore initiated on day 7 with concurrent ELISA analysis of AF-16 levels in blood immediately before and 30 min after each Salovum® administration (Fig 1b). A total of 5 doses were given. Like in the first patient, we observed that there was a retention of the solution in the stomach with increasing fluid volumes over time. ICP could not be controlled even though thiopental infusion was commenced (Fig. 1b, Table 1). The AF-16 ELISA analysis showed no statistically significant increase of AF-16 blood levels after nasogastric Salovum® administration (1.33 ± 0.04 before vs 1.35 ± 0.04 after, *p* = 0.65, *n* = 5 paired samples). Rectal administration of Salovum® was therefore initiated when ICP started to peak at levels above 30 mm Hg on day 9 with CT showing increased focal edema around the remaining contusions. Six doses of Salovum® were given via a rectal tube during 48 h with a statistically significant, transient reduction of ICP (Table 1). A single ELISA analysis of AF-16 levels in blood showed a value of 1.47 after Salovum® administration (30 min) compared with 1.38 immediately before administration. Due to the development of hydrocephalus, a sustained ICP control could not be achieved until the EVD was opened for continuous CSF drainage. The EVD could be removed on day 20 after the trauma, and the patient could be taken off from mechanical ventilation a week later. At 1-year control, he showed significant neurological sequelae with epilepsy and spasticity and needed full help with performing activities of daily living.

#### Lessons learned

Gastric retention after nasogastric administration of Salovum® was observed in similarity to the first patient without achieving control of ICP. The additional analysis of AF-16 levels in blood confirmed that the nasogastric route is not optimal for AF delivery in ventilated, critically ill patients due to gastroparesis with impaired emptying. Based on the observation of reduction in ICP in the course of rectal administration of Salovum®, this route seems to be more effective for delivery of AF. Additional blood tests are needed to confirm this presumption. AF has no effect on elevated ICP due to hydrocephalus.

### Patient no. 3 (Fig. 1c)

A 21-year-old female was involved in a bicycle accident where she was hit by a car without wearing a helmet—GCS 3 prior to intubation at the site of the accident. The patient was hemodynamically unstable, and her right pupil was slightly dilated and unreactive to light. Trauma CT showed signs of a general brain edema with obliterated sulci and compressed basal cisterns, as well as multiple punctate, supratentorial hemorrhagic lesions and a hematoma in the corpus callosum signifying diffuse axonal injury. The patient also had multiple fractures involving the pelvis and sacrum and her right leg. She received a Spiegelberg™ parenchymal probe showing ICP < 20 mm Hg which increased after external fixation of an open tibial fracture in her leg (Fig. 1c). Due to hemodynamic instability, rectal administration of Salovum® was initiated instead of thiopental infusion for treatment of the cerebral edema. A total of six doses were given rectally with a quick reduction of ICP to levels below 20 mm Hg (Fig. 1c). The ICP reduction was statistically significant and was sustained at levels < 20 mm Hg during most of the time of Salovum® administration (Table 1). Three AF-16 ELISA analyses all demonstrated increased values in blood 30 min after Salovum® administration (1.20, 1.26, and 1.24 before vs. 2.1, 1.9, and 2.0 after).

After termination of Salovum® administration, ICP started to rise on day 5. The Spiegelberg™ probe was exchanged to an EVD to allow intermittent drainage of CSF, and treatment with thiopental was initiated in an attempt to control ICP (Fig. 1c). EEG showed isoelectric activity. ICP peaked at values > 40 mm Hg with CT showing general brain swelling and progression of the hematoma in the corpus callosum. The patient developed atrial fibrillation which was electrically converted into sinus rhythm but later in the evening episodes of bradycardia occurred. The patient died due to cardiac arrest early in the morning on day 6 after the trauma.

#### Lessons learned

Even though the NICU management was optimized and ICP could be controlled during the course of Salovum® administration, the patient was critically ill and could not be saved due to the severe brain injuries, multiple fractures, and hemodynamic instability. Blood tests confirmed that AF in Salovum® is absorbed adequately after rectal administration. The administration of Salovum® by the rectal route needs to be extended in time with concomitant blood tests, using additional ELISA methods for consolidating the uptake of AF.

### Patient no. 4 (Fig. 1d)

A 30-year-old female had been exposed to a severe head trauma in a car accident. She was evaluated being GCS 7 at the scene of the accident prior to intubation. Trauma CT showed multiple thoracic injuries and a cervical fracture but no intracranial pathological findings requiring surgical intervention. Follow-up CT the day after the trauma showed development of multiple cerebral and cerebellar focal hypodense areas indicative of ischemia/infarction due to dissection of the left vertebral and the right internal carotid artery which was demonstrated with CT angiography [22, 25]. A Raumedic Neurovent-P intraparenchymal microtransducer was inserted intracranially for ICP monitoring, and the patient was transferred from the general intensive care unit to the NICU. Except for a temporary ICP elevation on day 4 to 5, with values exceeding 20 mm Hg due to the development of edema around the focal ischemic areas which required intermittent thiopental administration, ICP could be kept under control.

On day 9 after the trauma, ICP started to rise to pressures remaining above 20 mm Hg, with peaks up to 30–40 mm Hg, without any correlating alteration in the status or management of the patient (Fig. 1d). CT showed development of a more general brain swelling causing mass-effect with compression of basal cisterns and ventricles, and obliteration of cerebral sulci. An EVD was inserted for intermittent drainage of CSF which was applied without any lasting effect on the elevated ICP (Fig. 1d). Treatment with thiopental infusion was therefore initiated with increase up to doses of 4 mg/kg/h with induction of a moderate burst suppression pattern on EEG, but without achieving control of ICP. At this point, before a decompressive craniectomy was considered, it was decided to include the patient in the pilot study and to extend the rectal administration of Salovum® from 2 to 3 days. The patient received a total of ten doses.

**Fig. 2:**
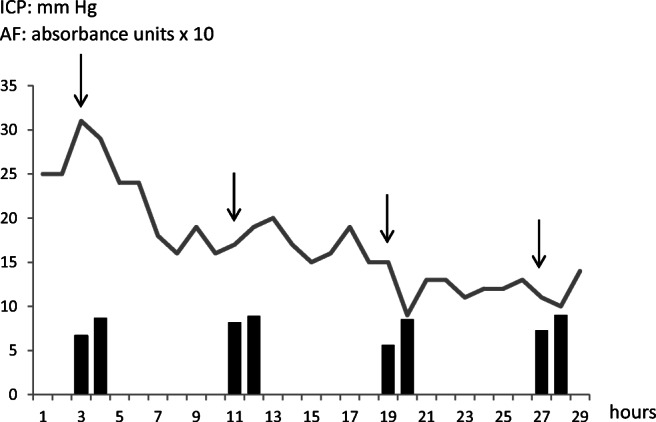
ICP during the time for the first four Salovum® administrations given every 8 h in patient 4. The bars below the ICP curve show antisecretory factor (AF) containing compleasome levels in blood (expressed in absorbance units × 10) from the ELISA analysis immediately before and 30 min after Salovum® administration. AF compleasome levels increase after each infusion of Salovum® (cf. Table 2)

**Fig. 3:**
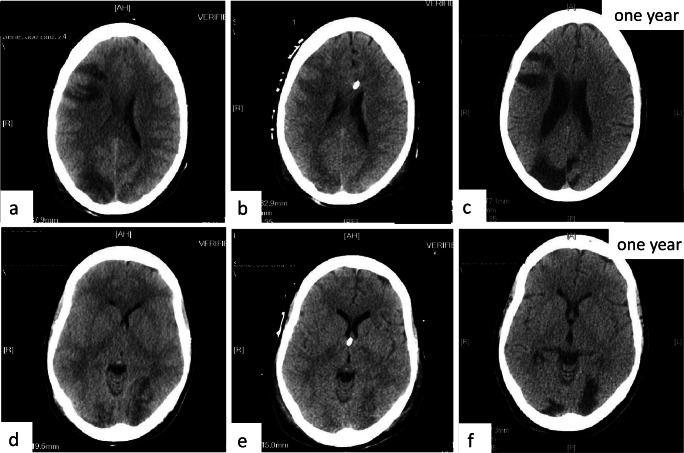
CT images showing two axial plane sections of the brain of patient 4 before and after AF treatment. **a**, **d** Day 9 after the trauma when ICP peaked at levels > 30 mm Hg, immediately before the insertion of an EVD (cf. Fig. 1d). Signs of general brain swelling causing a mass effect with obliterated cerebral sulci and compressed ventricles are present in addition to the focal, hypodense, ischemic areas. **b**, **e** Day 14, 70 h after the start of Salovum® administration. Sulci are visible and the compressed ventricles have opened up. The focal, hypodense areas have faded. **c**, **f** Follow-up 1 year after the trauma

A rapid suppression of ICP to levels < 20 mm Hg, which were sustained in the long term, was obtained within 4 h after the first rectal infusion of Salovum® (Figs. 1d and 2). Statistical analysis showed that ICP during the course of Salovum® administration was significantly reduced, compared with ICP during the 20 h preceding the addition of Salovum® (Table 1). A statistically significant increase of both AF-16 and AF containing compleasome in plasma was seen after Salovum® administration (Table 2, cf. Fig. 2). The thiopental infusion could be discontinued before termination of Salovum® treatment. CT scan 70 h after the start of Salovum® administration showed that the compressed basal cisterns and ventricles had opened up and the obliterated cerebral sulci had become visible again (Fig. 3).

**Table 2:** ELISA analyses of plasma samples immediately before and 30 min after start of rectal Salovum® administration in patient 4

ELISA	Before Salovum® infusion (n=10)	After Salovum® infusion (n=10)	p-value before *vs.* after Salovum® infusion (paired t-test)
AF-16**	0.947 ± 0.046*	0.989 ± 0.050	0.027
Compleasome	0.59 ± 0.046*	0.68 ± 0.045	0.045

*n* = number of plasma samples

*Values represent mean ± SEM expressed in absorbance units

**AF-16 absorbance is presented as inverted values

After conclusion of Salovum® administration, the cervical fracture was stabilized, and the patient could be taken off of mechanical ventilation. She was discharged from the NICU 28 days after the trauma and went eventually to rehabilitation. At 1-year control, the patient had a slightly impaired function with some spasticity in her left arm, a weakness with pain in the left hand, and a blurring of the lower vision fields in both eyes (cf. Fig. 3). The patient lived on her own, managing all activities of daily living.

No adverse events that could be attributed to AF treatment or the rectal approach for Salovum® administration were observed in any of the included patients.

## Discussion

This report describes the first steps to investigate the uptake, safety, and influence of AF for suppression of elevated ICP in patients with severe TBI. The parameters for optimal administration/uptake of Salovum® were established within the scope of a pilot study demonstrating an association between an increase of AF levels in blood after rectal administration of Salovum®, with a simultaneous reduction in ICP. In conformity with previous clinical studies investigating the antisecretory and anti-inflammatory actions of AF under various pathological conditions, no systemic side effects that could be attributed to Salovum® treatment were observed in any of the included patients [10, 20, 27, 28]. The outcomes suggest that AF as an add-on therapy may, directly or indirectly, have some suppressive influence on edema-induced, intracranial hypertension.

Salovum® is enriched with AF peptides, among them AF-16 [16], which in experimental animal models of focal brain injury has been demonstrated to exert a significant, suppressive effect on cerebral edema with pathologically elevated ICP, and to improve cognitive function [1, 6, 11]. AF-16 can bypass the blood-brain barrier after iv and intranasal administration and is detected both in cerebral tissue and CSF [1, 17]. In clinical settings, Salovum® has only been utilized orally in awake patients for treatment of various inflammatory conditions [10, 27, 28]. The uptake of AF for achieving a clinical response after oral administration is dependent on a functioning gastrointestinal passage [20]. In the first two patients in which Salovum® was delivered repeatedly via a nasogastric tube, there was an increased gastric retention of the solution due to gastrointestinal dysmotility which often is present in mechanically ventilated, critically ill patients, especially those with intracranial hypertension receiving opioids for analgesia and sedation [13, 18]. This observation is in line with the recent findings by Cederberg et al. investigating the effects of AF in patients with severe TBI after nasogastric administration of Salovum® [4]. The retention was stressful to the patients, and no obvious ICP reduction was observed (cf. Table 1). Therefore, in the second patient, we also tested to administer the solution rectally which gave a transient reduction of the elevated ICP (cf. Table 1). The ELISA analysis in the second and third patients showed that blood levels of AF-16 were increased after rectal but not after nasogastric Salovum® administration. Accordingly, the initial strategy for delivery of Salovum® was changed from a nasogastric to a rectal approach with a subsequent increase in plasma concentration of AF-16 which correlated with a statistically significant reduction in ICP. The strategy for rectal delivery was extended from 2 to 3 days in the fourth patient where blood levels of both AF-16 and AF compleasome were significantly increased after rectal Salovum® administration. Merged data from patients 2, 3, and 4 consolidated the statistically significant increase in blood levels of AF-16 after rectal Salovum® administration (*p* = 0.02, *n* = 14 paired samples).

The small number of included patients is a limitation of the present pilot study. The main purpose of the study was, however, met in that we could identify the optimal means for delivery of Salovum® in order to reduce pathologically elevated ICP in critically ill patients with severe TBI. This has not been investigated previously. Even though the observed reduction in ICP may in part be a consequence of the natural course of the TBI pathophysiology with gradual decrease of edema with time, or could reflect the accumulated effect of other NICU treatments, including thiopental, the influence of AF seems to be of importance based on (i) the correlation between the increase of AF levels in blood with an accompanying reduction in ICP; (ii) the rapid, substantial reduction of ICP to levels < 20 mm Hg within hours after start of rectal Salovum® administration which was observed in the two last patients who both presented with prominent cerebral edema; (iii) the demonstrated, statistically significant difference in mean ICP calculated from hourly registrations before and during the period of rectal administration of Salovum®, irrespective of whether or not the patients were under treatment with thiopental; (iv) the maintenance of ICP reduction at values < 20 mm Hg during most of the course of rectal Salovum® treatment. In addition, the outcomes in patient 2 suggested that AF has no effect on elevated ICP due to hydrocephalus. This observation is in line with previous findings on lack of efficacy in patients with idiopathic normal pressure hydrocephalus [9]. Thus, AF mainly seems to exert its effects on elevated ICP based on the occurrence of cerebral edema.

The molecular and cellular pathways that contribute to the development of posttraumatic brain edema are multifactorial and need to be more precisely defined in order to bring forth adequate therapies for targeting the edema [5, 8, 15, 24, 26]. Use of AF may offer a new means to further investigate and affect these events clinically without experiencing the negative side effects seen in treatment with barbiturates. Synthetic AF peptides, which can be administered iv for optimal uptake in a clinical setting, will be available in a near future. This will enable better characterization of AF kinetics with possibility to design randomized trials to further exploit potential AF effects on various types of brain edema.
